# Case report: Medulla oblongata and cervical cord reperfusion injury after intracranial vertebral artery angioplasty and stenting

**DOI:** 10.3389/fneur.2023.1097252

**Published:** 2023-05-05

**Authors:** Guiping Wang, Bo Zuo, Jia Jia, Jinlong Huang, Gangming Xi, Zhigang Yang

**Affiliations:** ^1^Department of Neurology, Shanghai Xuhui Central Hospital, Fudan University, Shanghai, China; ^2^Department of Neurosurgery, Zhongshan Hospital, Fudan University, Shanghai, China

**Keywords:** medulla oblongata, cervical cord, reperfusion injury, white cord syndrome, vertebral artery, angioplasty, stenting, stroke

## Abstract

**Background:**

White cord syndrome is an uncommon complication characterized by delayed neurologic deterioration with no other identified cause after spinal decompression surgery. Its etiology is attributed to spinal cord reperfusion injury. Here, we present the first case of an extended version of white cord syndrome, with concomitant involvement of the medulla oblongata and cervical cord reperfusion injury after intracranial vertebral artery angioplasty and stenting.

**Case presentation:**

A 56-year-old male suffered an ischemic stroke in the right anteromedial medulla oblongata. Angiography revealed bilateral vertebral artery stenosis in the intracranial segment. We performed elective left vertebral artery angioplasty and stenting. An intraoperative flow arrest in the left VA occurred and was stopped after the withdrawal of the catheter. Several hours after the operation, the patient developed occipital headache, back neck pain, dysarthria, and worsening left-sided hemiplegia. Magnetic resonance imaging revealed hyperintensity and swelling in the medulla oblongata and cervical cord, in addition to small medullary infarction. A digital subtraction angiography revealed intact vertebrobasilar arteries and patency of the left vertebral artery, left posterior inferior cerebellar artery, and implanted stent. We considered that the reperfusion injury had caused the complication. After treatment, the patient’s symptoms and neurologic deficits greatly improved. He achieved a favorable outcome at the 1-year follow-up, with normal intensity restored in the medulla oblongata and cervical cord on magnetic resonance imaging.

**Conclusion:**

Concomitant reperfusion injury in the medulla oblongata and cervical cord secondary to vertebral artery angioplasty and stenting is extremely rare. However, this potentially devastating complication requires early recognition and prompt treatment. Maintaining the antegrade flow during vertebral artery endovascular treatment is a precaution against reperfusion injury.

## 1. Introduction

White cord syndrome (WCS) is defined as an acute neurologic deterioration after spinal decompression surgery, mostly after cervical spine surgery. Typical magnetic resonance imaging (MRI) features include intramedullary hyperintensity on T2-weighted images representing cord edema, ischemia, swelling, and/or hemorrhage without any other extrinsic pathology (1). While reperfusion injury is its probable cause, WCS identification remains a diagnosis of exclusion (1, 2). Only one WCS case involving the medulla oblongata has been reported (3), and no report connecting WCS and endovascular treatment of the vertebral artery (VA) exists. Here, we report a case of delayed-onset medulla oblongata and cervical cord reperfusion injury in a patient exhibiting newly developed occipital headache, back neck pain, dysarthria, and worsening left-sided hemiplegia hours after successful left intracranial VA angioplasty and stenting. To our knowledge, this is the first report of simultaneous reperfusion injuries in the medulla oblongata and cervical cord related to VA endovascular treatment. This is also the first extended version of WCS, which is no longer limited to spinal cord lesions or spinal decompression surgery.

## 2. Case presentation

**Figure 1:**
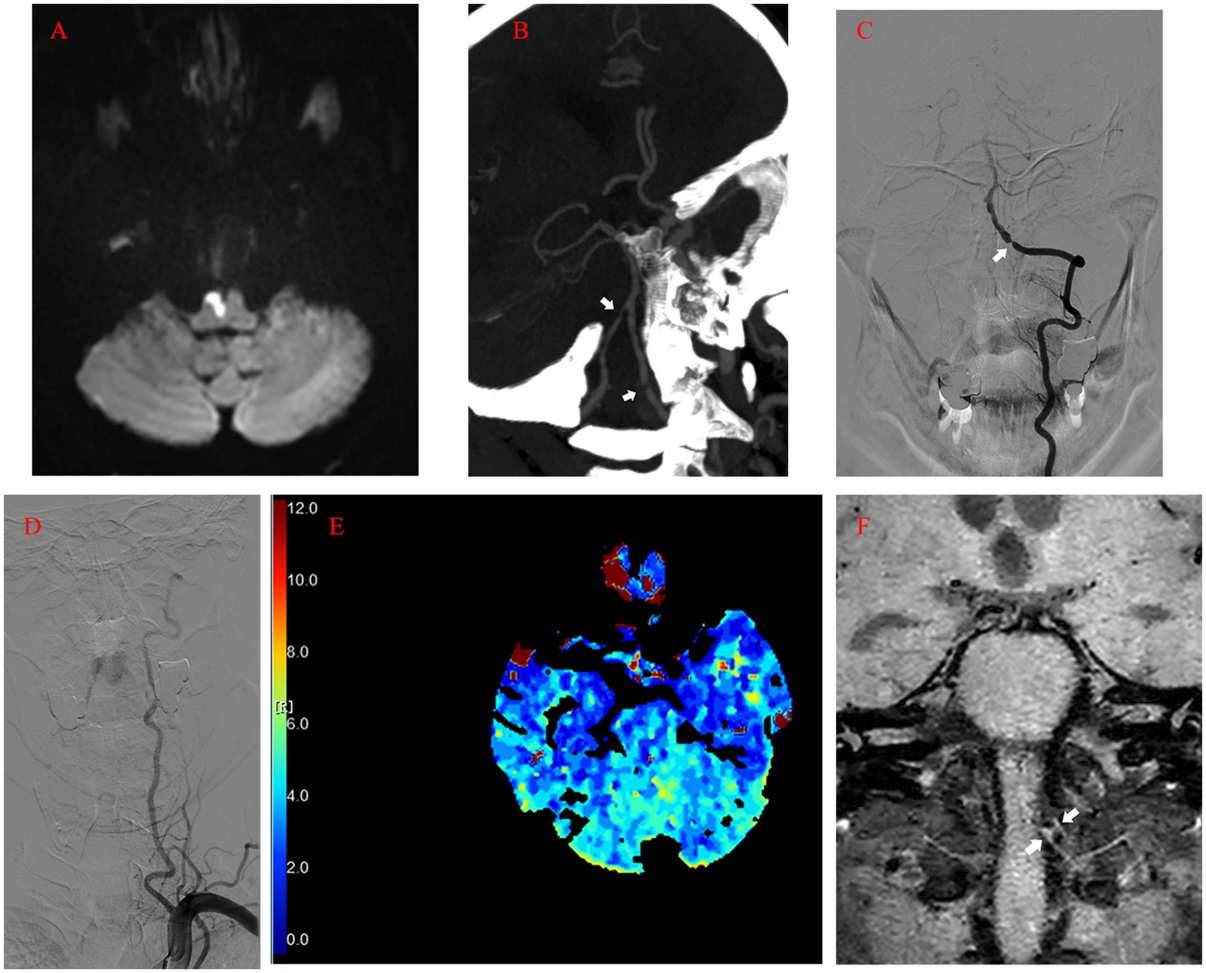
**(A)** Acute infarction of the right anteromedial medulla oblongata visualized by MRI (diffusion-weighted imaging). **(B)** Computed tomography angiography image showing the bilateral VA stenosis in the intracranial segment (arrow). **(C)** Left vertebral angiogram (anterior–posterior view) showing preocclusive stenosis of the left intracranial VA (arrow). **(D)** Tortuosity of the left VA revealed by angiogram. **(E)** Computed tomography perfusion showing the marked hypoperfusion in the brain stem and cerebella (Tmax). **(F)** High-resolution MRI showing atheromatous plaques (arrow) in the stenotic segment of the left VA (coronal view). MRI, magnetic resonance imaging; VA, vertebral artery; Tmax, Time to Max.

A 56-year-old male presented with worsening dizziness and left-sided hemiplegia overnight. The patient’s medical history included 10-year-long hypertension and type 2 diabetes. The physical examination on admission revealed grade 0 muscle strength in the upper left extremity, grade 3 muscle strength in the lower left extremity, and left-sided hypoesthesia (Table 1). MRI demonstrated a new-onset ischemic stroke in the right anteromedial medulla oblongata (Figure 1A) and an old hemorrhage in the right medial pons. Computed tomography angiography (CTA) revealed bilateral VA stenosis in the intracranial segment (Figure 1B). Later digital subtraction angiography (DSA) confirmed this result, demonstrating a preocclusive (> 90%) narrowing of the left intracranial VA and tortuosity of the left VA (Figures 1C,D). We also found marked hypoperfusion in the brain stem and cerebella by computed tomography perfusion (Figure 1E). High-resolution MRI revealed atheromatous plaques in the stenotic segment of the left VA (Figure 1F). The patient’s state had remarkably improved at discharge after treatment and rehabilitation.

**Table 1:** Timeline of clinical, imaging and procedural data.

First admission	Worsening dizziness and left-sided hemiplegia; PE: grade 0 muscle strength in the upper left extremity, grade 3 muscle strength in the lower left extremity, and left-sided hypoesthesia
Day 8	Cerebral angiography
Day 15	Discharged with grade 4 muscle strength in left extremities
Second admission	PE: grade 4+ muscle strength in left extremities
Day 6	Left vertebral artery angioplasty and stenting
4 h after procedure	Occipital headache and back neck pain
10 h after procedure	PE: dysarthria, left-sided hemiplegia and elevated blood pressure
Day 7	MRI: acute infarction of the left posterior medulla oblongata on DWI and newly developed hyperintensity in the left medulla oblongata on T2 and FLAIR Cerebral angiography
Day 11	Cervical spine MRI: hyperintensity and swelling in the cervical cord
Day 37	MRI: near-normal intensity in the medulla oblongata and cervical cord
Day 38	Discharged with grade 4+ muscle strength in left extremities
1 year	Clinical, MRI and Computed tomography angiography imaging follow up

PE, physical examination; MRI, magnetic resonance imaging; DWI, diffusion-weighted imaging; FLAIR, fluid-attenuated inversion recovery.

**Figure 2:**
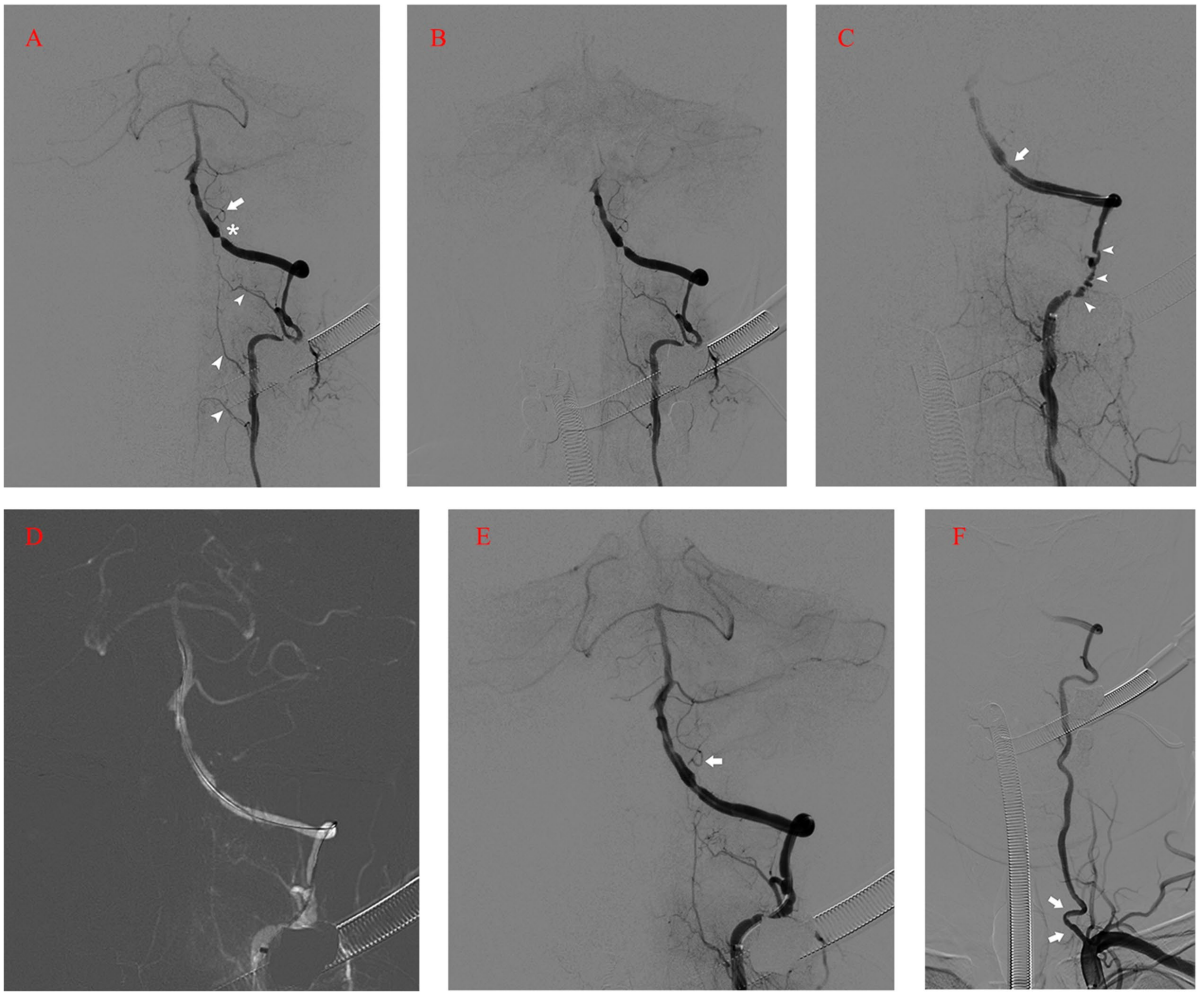
**(A)** Angiography with DAC in the distal V2 segment revealing severe stenosis (asterisk), dysplasia in the left PICA (arrow), and anastomosis supplying blood flow from the left VA to the cervical cord (arrowhead). **(B)** Left vertebral angiogram showing the venous phase of the intracranial anatomy and left VA flow arrest. **(C)** Post-angioplasty angiography revealing the improvement of the stenosis (arrow) and marked spasm of the left V3 segment (arrow-head). **(D)** Roadmap after intra-artery nimodipine showing the spasm mitigation process. **(E)** Control run after the deployment of Enterprise stent showing the patency of the left VA and PICA (arrow). **(F)** The final run showing the minor spasms in the initial segment of the left VA (arrow). DAC, Distal Access Catheter; PICA, Posterior Inferior Cerebellar Artery; VA, Vertebral Artery.

After 50 days of rehabilitation, he was brought in for endovascular treatment. Physical examination on the second admission showed grade 4+ muscle strength in left extremities. Preoperative MRI revealed no new ischemic stroke. Five days after admission, the patient’s left intracranial VA was recanalized under general anesthesia. A 90 cm 6F Neuronmax088 long sheath (Penumbra, Inc., Alameda, CA, USA) was introduced into the initial segment of the left subclavian artery. Angiography with a 115 cm 5F AXS Catalyst 5 distal access catheter (DAC) (Stryker Neurovascular, Michigan, USA) in the distal V2 segment revealed severe stenosis, dysplasia in the left posterior inferior cerebellar artery (PICA), and anastomosis supplying blood flow from the left VA to the cervical cord (Figure 2A). A concomitant clear visualization of the left VA and venous phase of the intracranial anatomy suggested that the DAC incompletely blocked blood flow (Figure 2B). A 2.25 × 9 mm Gateway balloon catheter (Stryker Neurovascular, Fremont, CA, USA) was delivered over a 300 cm synchro2 microguidewire (Stryker Neurovascular, Salt Lake, Utah, USA) to the site and slowly dilated to 7 atm. Post-angioplasty angiography revealed improvement of the stenosis and marked spasm of the left V3 segment (Figure 2C). The slow infusion of 1 mg of intra-artery nimodipine alleviated the spasms (Figure 2D). Next, a 4.5 × 14 mm Enterprise stent (Codman & Shurtleff, Inc., Raynham, MA, USA) was quickly and successfully implanted in the stenotic segment. The DAC was withdrawn immediately after the control run (Figure 2E). The final run showed only minor spasms in the initial segment of the left VA (Figure 2F). The patient showed no neurologic deterioration on awakening. Proper blood pressure was maintained using intravenous urapidil, while tirofiban was used to prevent acute stent thrombosis. Four hours later, the patient developed an occipital headache and back neck pain, mainly on the left side. Postoperative CT imaging revealed nothing significant.

**Figure 3:**
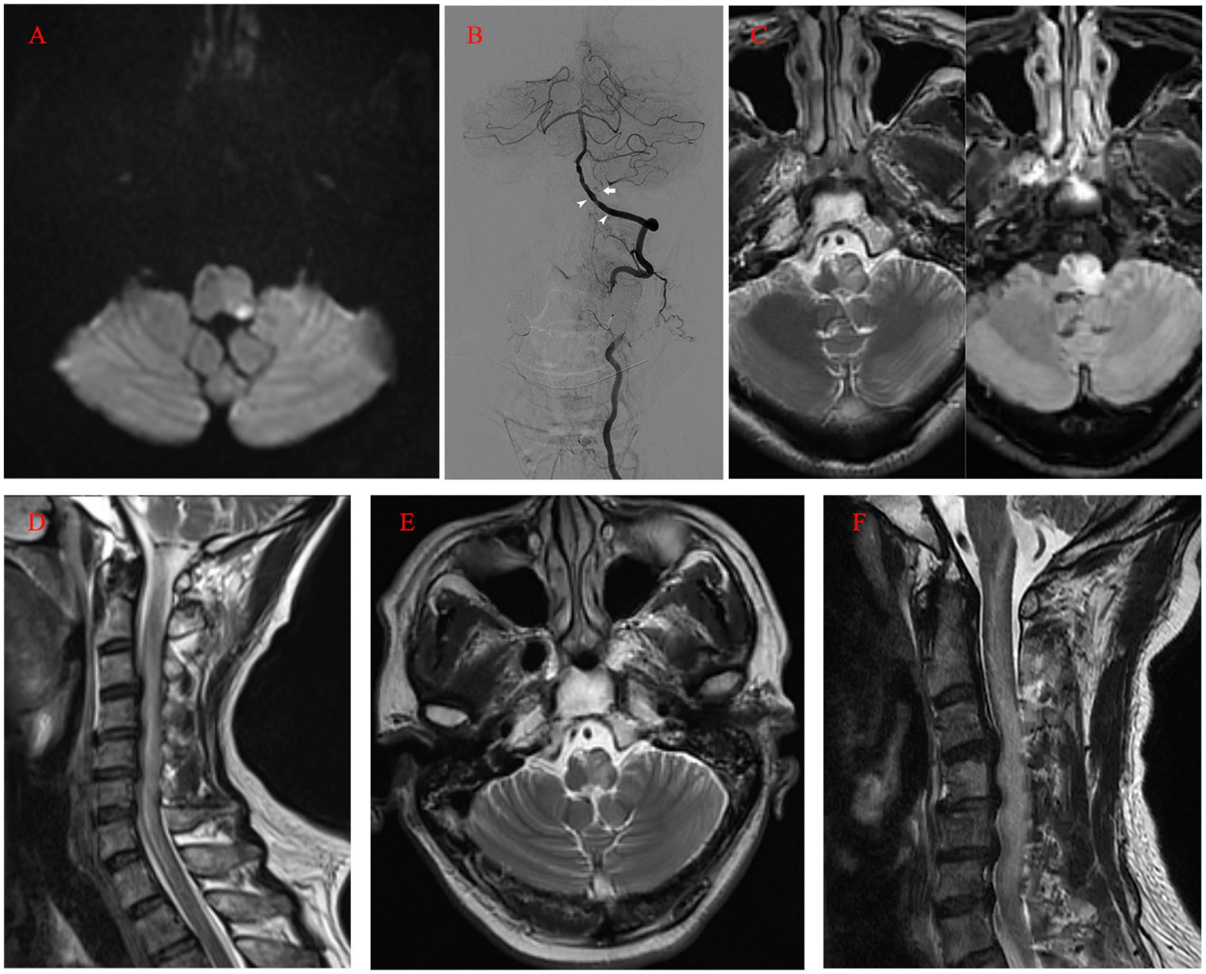
**(A)** MRI (diffusion-weighted imaging) on the first day after the operation showing the acute infarction of the left posterior medulla oblongata. **(B)** Postoperative angiogram showing the intact vertebrobasilar arteries and patency of the left VA, left PICA (arrow), and implanted stent (arrowhead). **(C)** MRI (T2 and FLAIR images) on the fourth day after the operation revealing profound hyperintensity in the left medulla oblongata. **(D)** Cervical spine MRI on the fifth day after the operation revealing similar hyperintensity and swelling in the cervical cord (T2). **(E, F)** Ten days after the operation, MRI still revealed hyperintensity in the medulla oblongata and cervical cord. MRI, magnetic resonance imaging; VA, Vertebral Artery; PICA, Posterior Inferior Cerebellar Artery; FLAIR, Fluid-Attenuated Inversion Recovery.

However, at midnight, symptoms worsened, accompanied by dysarthria, left-sided hemiplegia, and elevated blood pressure (around 170/100 mmHg) (Table 1). Again, CT imaging showed no marked changes, while MRI revealed acute infarction of the left posterior medulla oblongata on the next morning (Figure 3A). In addition to the new infarction, T2-weighted and fluid-attenuated inversion recovery (Flair)-weighted images showed newly developed hyperintensity in the left medulla oblongata. Subsequent DSA demonstrated intact vertebrobasilar arteries and patency of the left VA, left PICA, and implanted stent (Figure 3B). To regulate blood pressure, we added an intravenous infusion of esmolol. We used Tirofiban for another 24 h before switching to routine dual antiplatelet treatment with 100 mg aspirin and 75 mg clopidogrel per day. The patient received an intravenous injection of 40 mg methylprednisolone twice per day, along with glycerol fructose and edaravone injections. Three days later, T2 and Flair images showed more profound hyperintensity in the left medulla oblongata (Figure 3C). One day later, a cervical spine MRI (T2 images) revealed unexpected findings of similar hyperintensity and swelling in the cervical cord (Figure 3D). We added an intravenous infusion of mannitol to ameliorate medulla oblongata and cord edema and started early rehabilitation.

**Figure 4:**
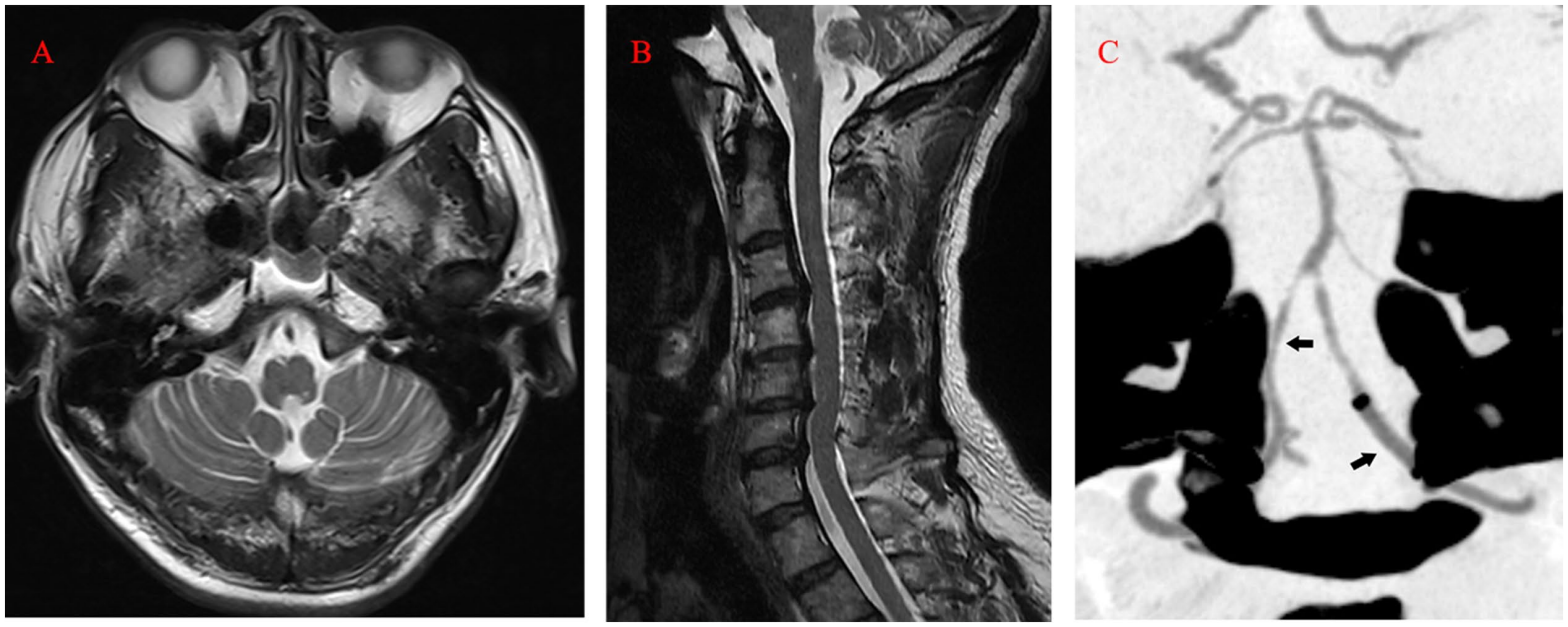
**(A, B)** At the 1-year follow-up, MRI (T2) showed normal intensity in the medulla oblongata and cervical cord. **(C)** Computed tomography angiography revealing the patency of both VAs (arrow). MRI, magnetic resonance imaging; VA, Vertebral Artery.

Ten days after the operation, MRI still showed marked hyperintensity in the medulla oblongata and cervical cord, while the patient’s symptoms and neurologic deficits gradually improved (Figures 3E,F). The final MRI before discharge showed near-normal intensity in the medulla oblongata and cervical cord, with only slight hyperintensity in the left region. At the 1-year follow-up, MRI showed normal intensity in the medulla oblongata and cervical cord, except for the old lesions (Figures 4A,B). Both VAs remained patent on the CTA (Figure 4C). The patient achieved a favorable outcome, with a modified Rankin scale score of 2. The physical examination revealed normal speech, grade 5 muscle strength in left limbs, and slightly increased muscle tone in the upper left limb.

## 3. Discussion

WCS is uncommon after cervical or thoracic spinal decompression surgery but potentially devastating (4). WCS is a possible explanation for delayed deterioration with no cause identified (5). We define our case as an extended version of WCS because the complication happened after VA endovascular treatment rather than after spinal surgery. The patient presented no new neurologic deficit until hours after the operation. Furthermore, to our best knowledge, the concomitant involvement of the medulla oblongata and cervical cord is rare. Using PubMed, we found one single case of WCS involving both the medulla oblongata and cervical cord. In that report, a cervical extramedullary metastatic ductal carcinoma led to cord compression and extended into both C3 neural foramina (3). Although the author did not mention why medulla oblongata was involved, we think that the removal of the tumor and decompression might have recanalized the VA and reperfused part of the medulla oblongata. The etiology of WCS is attributed to a sudden increase in blood supply to the spinal cord after decompression. The subsequent compromise of the blood–brain barrier and the blood-spinal cord barrier results in reperfusion injury (3). On the one hand, our case, in the absence of direct cord manipulation, demonstrates that inappropriate manipulation is not a causative factor of WCS in spinal decompression surgery (6). On the other hand, the underlying mechanism of our case needs further investigation due to the lack of surgical decompression.

Matsubara reported a case of ruptured VA dissecting aneurysm treated with internal trapping. The patient developed concomitant medulla oblongata and cervical cord infarction postoperatively, with a poor outcome. The author attributed the complication to the obstruction of perforating arteries supplying the medulla oblongata and ischemia of the spinal artery branches originating from the left VA (7). The upper medulla oblongata is supplied by a pair of anterior spinal arteries originating from the VA. The lateral region of the medulla oblongata is perfused by PICA or VA, while the posterior part is perfused by the posterior spinal artery, which originates from the VA or PICA (8). Tsuruta et al. found that intraoperative proximal flow arrest was performed in all four symptomatic infarction cases of VA dissecting aneurysm embolization. They believe that a long segmental flow arrest carries the risk of collateral circulation insufficiency and thromboembolism, resulting in medulla oblongata and cervical cord infarction (9). We used DAC to provide both steady proximal support and proximity to stenosis during the operation. However, blood flow was arrested in a long VA segment (Figure 2B) due to its tortuosity. Although we rapidly withdrew the DAC after the quick and necessary manipulations and restored blood flow (Figure 2E), reperfusion injury was initiated instead of complete infarction.

Therefore, in our case, all the branches mentioned above, supplying the left medulla oblongata and cervical cord, might have been affected due to the flow arrest in the left VA. It is likely that the temporary obstruction of perforating, anterior spinal, and posterior spinal artery branches arising from the left VA, resulting from the flow arrest by the DAC through tortuous vasculature, led to a short ischemia period and subsequent reperfusion injury. Notably, after the pass of the balloon catheter, we observed significant spasms in the V3 segment (Figure 2C). Although intra-artery nimodipine mitigated the spasms, this minor reperfusion process might have caused injury as well. The dual reperfusion may be the cause of our rare case. Either a part of the collateral network remained compromised and led to acute infarction of the left posterior medulla oblongata, or the long segmental flow arrest caused thromboembolism. The posterior spinal artery has rich collateral networks and a smaller risk of infarction (10). Although we did not perform a more complex spine MRI with diffusion-weighted imaging, we believe that the cervical cord underwent only a minor infarction based on the follow-up MRI (Figure 4B) and the patient’s quick recovery and good outcome. Maintaining antegrade flow during VA endovascular treatment is crucial to prevent intraoperative infarction and postoperative reperfusion injury. Although the flow arrest should have affected the left PICA, we found no infarction or reperfusion injury in the left cerebellar on postoperative MRI because the major blood supply of the patient’s left cerebellar came from the right PICA. Tanoue et al. evaluated anatomical variations of perforating arteries from VA using three-dimensional DSA. They found that non-PICA VAs give off a larger number of perforators than other types, indicating a higher risk of ischemic stroke with trapping (11). In our case, the left PICA was hypoplastic and, similar to non-PICA VAs, had a potentially higher risk of infarction and reperfusion injury secondary to VA flow arrest.

Cerebral hyperperfusion syndrome (CHS) after angioplasty or stenting for intracranial artery stenosis is a rare but severe complication. CHS is characterized by headaches, seizures, and neurologic deficits not caused by cerebral ischemia. Since it impairs cerebral autoregulation, CHS can present as cerebral edema, hemorrhage, or subarachnoid hemorrhage (12). In our case, the postoperative hyperintensity region was not located exactly downstream of the VA stenosis. If CHS had been the cause of the complication, the downstream regions such as the pons, midbrain, left cerebella, and left occipital lobe should have been affected.

The precise mechanism of WCS remains unclear but probably involves oxygen free radicals, lipid peroxidation, inflammation, and specific signal cascades (13–16). In our case of extended WCS, intraoperative flow arrest and restoration may have triggered a similar pathophysiological process. Therefore, we managed the complication based on the mechanism mentioned above. In an attempt to mitigate medulla oblongata and cervical cord edema, we administered methylprednisolone. A high dose might have enhanced early recovery by inhibiting inflammation and lipid peroxidation (17, 18). Nevertheless, considering gastrointestinal bleeding and hyperglycemia risks, we preferred a low methylprednisolone dose. Edaravone, a free radical scavenger, may have played a role in the patient’s recovery as well. Suzuki et al. reported that edaravone protected against spinal cord ischemia–reperfusion injury in a rabbit model by reducing free radical species levels (19). Edaravone also protects against cerebral reperfusion injury *via* oxidative stress and a specific signal pathway involving mitochondrial dysfunction and apoptosis (20). Previous reports recognize physical therapy as an important treatment modality for injury and weakness (2). In our case, because the patient’s respiratory and cardiac rhythm remained in the normal range despite the swelling of the left medulla oblongata, we started inpatient physical therapy four days after the complication occurrence. Next, long-term outpatient rehabilitation brought further functional improvement.

## 4. Conclusion

Concomitant reperfusion injury in the medulla oblongata and cervical cord is extremely rare after VA angioplasty and stenting. Nevertheless, clinicians should be fully aware of this rare but potentially devastating complication and maintain antegrade flow during VA endovascular treatment. Moreover, this complication should be added to informed consent forms, for precaution.

## Data availability statement

The original contributions presented in the study are included in the article/supplementary material, further inquiries can be directed to the corresponding author.

## Ethics statement

Written informed consent was obtained from the participant/patient(s) for the publication of this case report.

## Author contributions

GW: manuscript writing. GW, BZ, and ZY: operation. GW, JH and JJ: data acquisition. GX and ZY: review and editing. All authors contributed to the article and approved the submitted version.

## Conflict of interest

The authors declare that the research was conducted in the absence of any commercial or financial relationships that could be construed as a potential conflict of interest.

## Publisher’s note

All claims expressed in this article are solely those of the authors and do not necessarily represent those of their affiliated organizations, or those of the publisher, the editors and the reviewers. Any product that may be evaluated in this article, or claim that may be made by its manufacturer, is not guaranteed or endorsed by the publisher.
